# Markedly decreasing azithromycin susceptibility of *Neisseria gonorrhoeae*, Germany, 2014 to 2021

**DOI:** 10.2807/1560-7917.ES.2021.26.31.2100616

**Published:** 2021-08-05

**Authors:** Regina Selb, Susanne Buder, Sandra Dudareva, Thalea Tamminga, Viviane Bremer, Sebastian Banhart, Dagmar Heuer, Klaus Jansen

**Affiliations:** 1 Unit 'HIV/AIDS, STI and Blood-borne Infections', Department of Infectious Disease Epidemiology, Robert Koch Institute, Berlin, Germany; 2 Unit 'Sexually Transmitted Bacterial Infections', Department of Infectious Diseases, Robert Koch Institute, Berlin, Germany

**Keywords:** *Neisseria gonorrhoeae*, antimicrobial resistance, AMR, azithromycin, minimum inhibitory concentration, MIC

## Abstract

We monitored antimicrobial susceptibility developments of *Neisseria gonorrhoeae* in Germany from January 2014 to May 2021. The proportion of isolates with azithromycin minimum inhibitory concentrations above the epidemiological cut-off increased substantially, from 1.3% in 2014 to 12.2% in 2020. Preliminary data from 2021 showed a further rise (January to May: 20.7%). Therefore, azithromycin as part of the recommended dual therapy in Germany for non-adherent patients is challenged. Antimicrobial susceptibility testing in clinical practice is crucial and continuous susceptibility surveillance indispensable.

The German national surveillance system on *Neisseria gonorrhoeae* (NG)-antimicrobial resistance (AMR) has been in place at the Robert Koch Institute (RKI) since 2014 [1]. Using susceptibility data of NG, we monitored the dynamics of the proportions of resistant isolates and of minimum inhibitory concentrations (MIC) in Germany from January 2014 to May 2021 to inform treatment guidelines.

## Epidemiological data and isolates

Between January 2014 and May 2021, a total of 87 diagnostic laboratories throughout Germany sent 3,253 vital *Neisseria gonorrhoeae* (NG) isolates together with respective epidemiological data to the RKI and the national reference laboratory for NG. The number of isolates collected increased over time. The male to female ratio was 9:1 (range over years: 6:1–12:1) over the observed period. The median age was 33 years (interquartile range (IQR): 26–44) for men and 29 years (IQR: 23–41) for women. For men, the most common swab regions were the urethra (87.4%; n = 2,472) and the rectal/anal region (4.7%; n = 132). For most NG samples from women, the isolation sites cervix (51.3%; n = 164) and vagina (22.8%; n = 73) were reported.

## Antimicrobial resistance patterns

**Figure 1:**
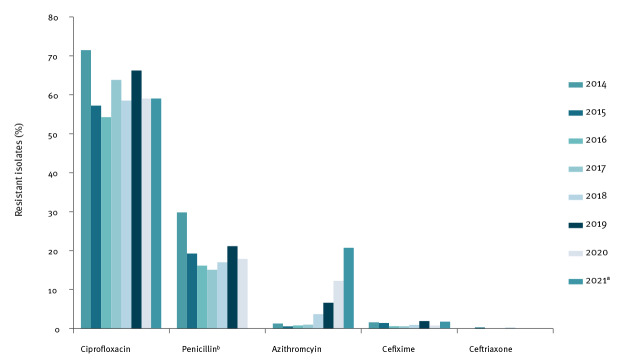
Proportion of antibiotic-resistant *Neisseria gonorrhoeae* isolates above ECOFF, Germany, 2014–2021 (n = 3,253)

ECOFF: epidemiologic cut-off; EUCAST: European Committee on Antimicrobial Susceptibility Testing.

^a^ Preliminary data for 2021 (January to May).

^b^ Penicillin was not measured in 2021.

We analysed the isolates for susceptibility to azithromycin, cefixime, ceftriaxone, ciprofloxacin and penicillin using E-test as described [1], without changes to the standard protocol. Quality assessments using World Health Organization (WHO) control strains were done every week [2]. For susceptibility interpretation, we applied the currently valid Clinical Breakpoint Table from the European Committee on Antimicrobial Susceptibility Testing (EUCAST) (v. 11.0) [3]. We noted a strong increase in the proportion of NG isolates with azithromycin minimum inhibitory concentrations (MIC) > 1 mg/L, above the epidemiological cut-off (ECOFF), between 2014 and 2020 in Germany (Figure 1). While in 2014, 1.3% (n = 4) and in 2015, 0.5% (n = 2) of isolates displayed an azithromycin MIC of > 1 mg/L, this proportion increased to 12.2% (n = 64) in 2020. Preliminary data of 2021 showed a continuation of this trend (January to May 2021: 20.7%). Proportions of ciprofloxacin- and penicillin-resistant isolates remained high, by year ranging from 54.3% to 71.2% for ciprofloxacin and from 15.1% to 29.8% for penicillin. We observed cefixime-resistant isolates in all years, with the highest proportion of 1.9% (n = 9) resistant isolates in 2019. Only one ceftriaxone-resistant isolate each was observed in 2015 and 2018, both with MIC values of 0.19 mg/L.

Resistance according to EUCAST v.11.0 [3].

## Development of azithromycin minimum inhibitory concentrations

**Figure 2:**
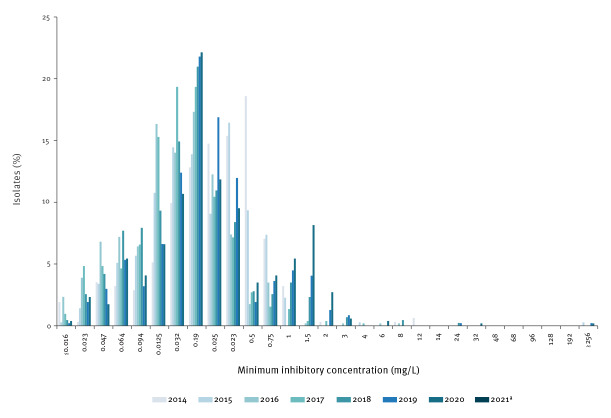
Distribution of *Neisseria gonorrhoeae* minimum inhibitory concentrations for azithromycin, Germany, 2014–2021 (n = 3,253)

^a^ Preliminary data for 2021 (January to May).

We were interested in a more detailed analysis of the development of azithromycin MIC from 2014 to 2020 and in the first months of 2021. While we observed a MIC50 of either 0.125 or of 0.19 for each year, the MIC90 shifted to higher values particularly between 2016 and 2021 (Table). Looking at the MIC values in detail (Figure 2), we found a noticeable population of isolates with MIC in the range of 0.38–1 mg/mL for the years 2014 and 2015. From 2016 onwards, we observed an increase in the proportion of isolates with MIC values between 0.19 and 2 mg/mL. To analyse this further, we divided the azithromycin MIC values in a total of five categories (Table). The first category, 0.016–0.25 mg/L, included all values regarded as sensitive for the whole observation period. The second category, 0.38–1 mg/L included values regarded as ‘intermediate’ or ‘resistant’ by EUCAST until 2019 but are categorised below ECOFF according to the currently valid standard. Values now considered above ECOFF (> 1 mg/L) were divided in three further categories.

**Table:** Number and proportion of *Neisseria gonorrhoeae* isolates in five MIC categories for azithromycin, Germany, 2014–2021 (n = 3,253)

	2014		2015		2016		2017		2018		2019		2020		2021^a^	
	n	%	n	%	n	%	n	%	n	%	n	%	n	%	n	%
≤ 0.016–0.25 mg/L	170	54.5	226	64.0	445	86.6	446	86.3	339	79.0	334	71.4	336	65.2	87	60.0
0.38–1 mg/L	138	44.2	125	35.4	65	12.7	66	12.8	74	17.3	103	22.0	116	22.6	28	19.3
1.5–4 mg/L	1	0.3	1	0.3	2	0.4	5	1.0	13	3.0	29	6.2	59	11.5	30	20.7
6–192 mg/L	3	1.0	0	0.0	2	0.4	0	0.0	3	0.7	1	0.2	3	0.6	0	0.0
≥ 256 mg/L	0	0.0	1	0.3	0	0.0	0	0.0	0	0.0	1	0.2	1	0.2	0	0.0
**Total isolates**	**312 **		**353**		**514 **		**517**		**429 **		**468 **		**515 **		**145 **	
MIC50	0.19		0.125		0.125		0.125		0.19		0.19		0.19		0.19	
MIC90	0.5		0.5		0.38		0.38		0.5		1		1.5		1.5	

MIC: minimum inhibitory concentration.

^a^ For 2021, preliminary data are shown for the months January to May.

In addition, MIC50 and MIC90 (in mg/L) for azithromycin are shown for each year. MIC50 and MIC90 are defined as the minimum concentration at which growth of 50% and 90% of the isolates is inhibited.

We found a marked increase in the proportion of isolates in the category above the ECOFF with MIC values between 1.5 and 4 mg/L during the entire period studied (Table); this distribution was statistically significant (Spearman’s rho: p = 0.02). The proportion of isolates just below the ECOFF (0.38–1 mg/L) decreased sharply from 2014 to 2016 and then increased until 2020. Preliminary data for 2021 showed a slight drop in this category. Single isolates with MIC values > 256mg/L were detected in 2015, 2019 and 2020.

## Discussion 

The emergence and spread of NG-AMR in Europe and worldwide poses a serious threat to the treatment and control of gonorrhoea [4-6]. In Germany, NG isolates with azithromycin MIC values above the ECOFF increased considerably from 2014 to 2020, with a pronounced rise in 2020. First data for the year 2021 underline the trend of decreasing azithromycin susceptibility; however, data are preliminary and have yet to be confirmed. At the same time, ceftriaxone remains effective for the treatment of gonorrhoea in Germany. 

Dual therapy consisting of azithromycin and ceftriaxone is the standard treatment recommended for gonorrhoea in Germany [7]. Because of the recent development of reduced azithromycin susceptibility, ceftriaxone monotherapy is preferred in the new German treatment guideline from December 2018 in well-controlled settings if adherence (adherence to therapy and control of cure) can be guaranteed [7]. The same approach is recommended in the current European guideline [8]. Dual therapy was introduced to mitigate a possible development of resistances, especially against ceftriaxone, and to target possible co-infections with *Chlamydia trachomatis* and *Mycoplasma genitalium* [9]. According to our current observations and data described by others, we may not be fully able to rely on azithromycin as a therapeutic agent for the treatment of gonorrhoea [4,10,11]. Antimicrobial susceptibility testing of all isolates in clinical practice and follow-up of the patients including test of cure are highly recommended and indispensable in particular for cases where monotherapeutic treatment with ceftriaxone is applied in accordance with the currently valid German guideline. Also, coinfections with *C. trachomatis* and *M. genitalium* should be checked and treated appropriately to avoid further AMR development. 

NG with high-level azithromycin resistance (> 256 mg/L) have been reported from several European countries and worldwide, and sustained transmission of these isolates has been described [4,10,12-16]. Interestingly, these isolates remain rare in Germany. In contrast, our data show that the increase in isolates with MIC values above the ECOFF for azithromycin concerns in particular isolates with MIC values in the range of 1–4 mg/L. Furthermore, starting from 2016, we observed a shift within the population of sensitive isolates to higher MIC values just below ECOFF (> 0.25–1 mg/L), isolates that were considered as intermediate or resistant until 2019. One possible explanation for the high proportions of isolates with MIC values in this range in the early project period (2014–2015) might be commonly applied monotherapeutic azithromycin treatment at the time. The first German guideline recommending dual therapy for the treatment of gonorrhoea was published in 2013; however, dual therapy was most probably fully implemented in clinical practice only after a transition phase of several years. In addition, a sampling effect in the establishing phase of the surveillance project might have played a role. 

Our data show that the population of isolates with increased azithromycin MIC values can be distinguished from the Gaussian MIC distribution of sensitive isolates, but these populations overlap partly. We could recently show that in 2018 in Germany, the increase in these isolates with MIC values around 1 mg/L can be attributed to the expansion of a clonal line harbouring a mosaic *mtr* locus acquired from commensal *Neisseria* species [17]. Since these isolates with acquired resistance also display MIC values < 1.5 mg/L, the establishment of an azithromycin resistance cut-off value (RCOFF) between 0.75 and 1 mg/L may be a useful complement to the ECOFF [18]. Taken together, NG-AMR surveillance is an important tool to detect critical changes in antibiotic susceptibility of NG in a timely manner. Further analyses on the genomic level are important to characterise population dynamics of isolates with reduced azithromycin susceptibility in 2019 and 2021.

## Conclusion

We observed a substantial increase in NG-isolates with azithromycin MIC values > 1 mg/L between January 2014 and May 2021. This could challenge the currently recommended dual therapy consisting of ceftriaxone and azithromycin in the near future. To this end, consistent antimicrobial susceptibility testing and control of cure are inevitable. 
