# Erratum for Euro Surveill. 2021;26(31)

**DOI:** 10.2807/1560-7917.ES.2021.26.35.210902e

**Published:** 2021-09-02

**Authors:** Eurosurveillance editorial team

**Affiliations:** 1 European Centre for Disease Prevention and Control (ECDC), Stockholm, Sweden

The title of Figure 1 in the article ‘Markedly decreasing azithromycin susceptibility of Neisseria gonorrhoeae, Germany, 2014 to 2021’ by Selb et al., published on 5 August 2021 has been corrected to read “Proportion of antibiotic-resistant Neisseria gonorrhoeae isolates/isolates above ECOFF” rather than “Proportion of antibiotic-resistant Neisseria gonorrhoeae isolates above ECOFF” as originally published. This change was made on 30 August 2021. 

